# Short- and long-term effectiveness of a three-month individualized need-supportive physical activity counseling intervention at the workplace

**DOI:** 10.1186/s12889-016-3965-1

**Published:** 2017-01-09

**Authors:** Anass Arrogi, Astrid Schotte, An Bogaerts, Filip Boen, Jan Seghers

**Affiliations:** 1 Department of Kinesiology, KU Leuven, Tervuursevest 101, 3001 Leuven, Belgium; 2 Faculty of Kinesiology and Rehabilitation Sciences, KU Leuven, Tervuursevest 101, 3001 Leuven, Belgium

**Keywords:** Physical activity promotion, Intervention, Worksite, Sedentary time, Mediation

## Abstract

**Background:**

The objective of the present study was to evaluate the short- and long-term intervention and mediation effects of a 3-month individualized need-supportive physical activity (PA) counseling intervention on employees’ PA and sedentary behavior.

**Methods:**

Insufficiently active employees (*n* = 300; mean age 42 ± 9 years; 78% female) were recruited from a large pharmaceutical company in Flanders, Belgium. A quasi-experimental design was used in which the intervention group (*N* = 246) was recruited separately from the reference group (*N* = 54). Intervention group participants received a 3-month behavioral support intervention, which consisted of two one-hour face-to-face counseling sessions and three follow-up counseling contacts by e-mail or telephone at weeks three, six and nine. PA counseling, delivered by qualified PA counselors, aimed to satisfy participants’ basic psychological needs for autonomy, competence, and relatedness. Reference group participants did not receive individualized PA counseling. Outcome measures included objectively assessed and self-reported PA and sedentary time and psychological need satisfaction. Assessments were held at baseline, immediately after the intervention (short-term) and 6 months post-intervention (long-term). Mixed model analyses and bootstrapping analyses were used to determine intervention and mediation effects, respectively.

**Results:**

The intervention group increased weekday daily steps both in the short- and long-term, while the reference group showed reductions in daily step count (ES = .65 and ES = .48 in the short- and long-term, respectively). In the short-term, weekday moderate-to-vigorous PA increased more pronouncedly in the intervention group compared to the reference group (ES = .34). Moreover, the intervention group demonstrated reductions in self-reported sitting time during weekends both in the short- and long-term, whereas the reference group reported increased sitting time (ES = .44 and ES = .32 in the short- and long-term, respectively). Changes in perceived autonomy and competence need satisfaction mediated the long-term intervention effects on daily step count.

**Conclusions:**

A 3-month individualized need-supportive PA counseling intervention among employees resulted in significant and sustained improvements in weekday daily step count and in decreased self-reported sitting during weekends. Our findings contribute to the growing evidence of the long-term effectiveness of need-supportive PA counseling.

**Trial registration:**

ClinicalTrials.gov NCT01759927. Registered December 30, 2012.

**Electronic supplementary material:**

The online version of this article (doi:10.1186/s12889-016-3965-1) contains supplementary material, which is available to authorized users.

## Background

Physical inactivity is considered to be a global health concern as it is ranked among the five leading risk factors for global all-cause mortality [1, 2]. In fact, recent self-reported physical activity (PA) data showed that more than one third of European adults did not meet contemporary PA recommendations [3] (i.e., either 30 min of moderate-intensity PA on five or more days a week or 20 min of vigorous-intensity PA on three or more days a week) [4]. As insufficiently active individuals are often unaware of their inactive lifestyle [5], lifestyle PA interventions have been developed to stimulate a more physically active lifestyle, for example by targeting awareness among subgroups of populations [6–8]. Insufficiently active employees represent one of the target subgroups of these interventions, especially in view of the progressive increase in the prevalence of sedentary PA occupations [9].

The workplace offers a great setting to promote PA because of the possibility to reach a substantial proportion of insufficiently active employees and the large amount of time adults spend at their work [10–12]. Recently, ‘a lack of time’ has been categorized as the most commonly cited barrier to exercise and/or engage in PA among European adults [3]. Arising from this perceived time constraint, the majority of insufficiently active employees experience difficulties on how to integrate a regular PA pattern into their daily life.

A meta-analysis revealed an overall ‘modest positive effect’ of workplace PA interventions on PA behavior [13]. Workplace PA interventions were recently characterized into three subcategories: physical activity/exercise interventions, counseling interventions and health promotion interventions [11]. Whereas the first category mainly focused on exercise engagement (e.g., exercise classes), the latter two intervention categories generally focused on lifestyle PA, (i.e., a daily accumulation of self-selected leisure, occupational or household activities that are at least of moderate intensity) [14]. Examples of interventions targeting lifestyle PA include individualized behavior change programs in which participants are encouraged to incorporate PA of moderate intensity into their daily routine (e.g., active transportation to and from work) [14, 15].

Lately, the need for more theory-driven PA interventions has been emphasized [11]. Following the previously mentioned ‘lack of time’ barrier, a perceived ‘lack of motivation’ is considered to be the second most reported barrier to exercise and/or engage in regular PA [3]. The most widely used comprehensive theory that targets and facilitates human motivation is the Self-Determination Theory (SDT) [16]. The central tenets proposed by SDT refer to the support of individuals to develop more autonomous and internalized forms of motivation (instead of externally-controlled extrinsic motivation) [16]. Interventions based on SDT tend to create a need-supportive environment in which individuals’ behavior change can be facilitated through satisfaction of three basic psychological needs: 1) the need for autonomy refers to feeling volitional in making informed and well-considered decisions; 2) the need for competence refers to feeling capable of achieving desired outcomes; and 3) the need for relatedness refers to feeling mutually connected to important others [16, 17].

The SDT has been driven interventions in multiple distinct contexts, including weight loss [18], smoking cessation [19] and diet regulation [20]. As adults tend to lose motivation towards exercise and PA, SDT-based PA interventions have been developed and implemented [21]. It has been shown that a need-supportive environment has the potential to facilitate and sustain PA behavior change (e.g., [22–26]). Based on the abovementioned advantages of workplace interventions, principles of SDT could inform and guide workplace PA interventions.

To the best of our knowledge, only one previous study has evaluated an SDT-based PA counseling intervention that was delivered at the workplace [27]. In a randomized controlled trial among insufficiently active university employees, Van Hoecke et al. found significant positive long-term effects of a 4-month need-supportive PA coaching intervention on moderate, vigorous and total PA [27]. The present study elaborates on that intervention by implementing additional self-monitoring tools including pedometers and PA diaries. Furthermore, the present study differs from the study by Van Hoecke et al. in terms of the additional intervention focus on sedentary behavior (in our intervention), the intervention setting (i.e., profit organization in our intervention versus university environment), and the assessment of PA levels (i.e., combined objective and subjective assessments in our study versus self-report only).

Taken together, the present need-supportive PA counseling intervention targets the two most frequently cited barriers to engage in PA (i.e., lack of time and motivation) by providing tailored PA counseling at the workplace and by creating a need-supportive environment intended to support employees in maintaining a regular PA pattern.

The PA counseling intervention aimed to satisfy the employees’ three basic psychological needs outlined by SDT. Examples of this approach include facilitating rather than prescribing PA (need for autonomy), supporting participants to implement their intention(s) to become physically active (need for competence) and encouraging participants to perform their PAs with colleagues (need for relatedness) [28, 29].

To this aim, the main objective of the current study was to evaluate the short- and long-term effectiveness of a 3-month individualized need-supportive PA counseling intervention at the workplace on PA and sedentary behavior. It was hypothesized that, compared to the reference group, the intervention group would show a more pronounced increase in PA behavior, and a decrease in sedentary time, both in the short- (immediately after the intervention) and long-term (6 months post-intervention) (hypothesis 1).

Besides examining the intervention effects on PA behavior, it is also important to evaluate the underlying mechanisms that explain the expected intervention effects. Previous PA intervention studies found mediating effects of self-efficacy [30], social support [31] and decisional balance (i.e., weighing PA benefits and barriers) [32], all arising from different theories and tested among adults and children [33, 34]. The current intervention aimed to satisfy participants’ psychological needs. Therefore, the second objective of the present study was to evaluate the mediation effects of psychological need satisfaction on changes in PA behavior. Because the intervention intended to influence participants’ psychological need satisfaction, we expected that the short- and long-term changes in PA behavior would be mediated by changes in psychological need satisfaction (hypothesis 2).

## Methods

### Participant recruitment and study design

Study participants were recruited from a large pharmaceutical company located in Flanders, the northern Dutch-speaking part of Belgium. The company employs over 3800 employees working across five worksites. Participants (*n* = 300) were recruited from two of the five worksites in two time waves, representing the first half year of 2013 and 2014, respectively. All employees of the company were invited by e-mail to attend an information session in which the purpose, organization, duration, benefits and registration of the PA intervention program was clarified. Eligible employees who were interested could subscribe to enroll in the intervention by an online registration tool.

The online registration tool consisted of a company’s health risk assessment (HRA) tool in which employees indicated the number of days and the accumulated number of minutes (in bouts of 10 min) during which they participated in moderate- and/or vigorous-intensity PA (MVPA) [35]. More specifically, they were asked on how many days, over the last thirty days, they engaged in activities of moderate and vigorous intensity (separate questions for each intensity). Subsequently, respondents were asked to quantify the number of minutes they accumulated on an average day. To calculate the equivalent combination of MVPA, minutes of vigorous activity were weighted by two to account for their greater intensity. Employees who were classified as being insufficiently active (i.e., not meeting the health-enhancing PA recommendations [4]) were invited to participate in the PA intervention program.

Throughout the period of baseline measurements, the PA intervention program was promoted by means of internal communication (distributed by the Environment, Health and Safety office) including e-mails to all employees, leaflets, posters, and screen-based announcements.

Participants were measured using a quasi-experimental design, in which intervention group participants (*n* = 246) were recruited separately from participants in the reference group (*n* = 54). Both groups were recruited from the same two worksites and during the same recruitment period. The reference group was recruited by means of a general call for participants who were interested in monitoring their activity pattern and assessing their physical health.

### Procedure

Participants read and signed an informed consent and all procedures were approved by the local Medical Ethics Committee of the KU Leuven, Belgium (s54788). At baseline, participants of the intervention group were screened on contraindications to engage in PA using the Physical Activity Readiness Questionnaire (PAR-Q) [36]. Participants were advised to visit a physician if they answered ‘yes’ to one or more of the PAR-Q items. They were, however, allowed to participate in the PA counseling intervention.

#### Intervention

**Fig. 1:**
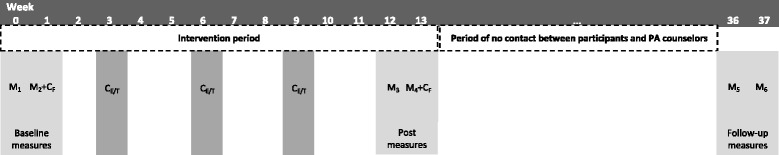
Time frame of the PA counseling intervention. Note: M = measurement session; C_F_ = face-to-face counseling session; C_E/T_ = e-mail/telephone counseling session

The 3-month intervention consisted of nine contact moments between participants and PA counselors (*n* = three): six face-to-face contact moments and three contact moments by e-mail or telephone (Fig. 1). Two of the six face-to-face contacts were explicitly planned as face-to-face counseling sessions, while the other four face-to-face sessions were mainly considered as measurement sessions. In addition, the three e-mail or telephone contact moments included PA counseling content and were part of the PA counseling (Fig. 1). The three PA counselors held Master degrees in Kinesiology, were educated in health-related PA counseling and were specialized in SDT-based counseling.

##### Intervention group

The initial session included an introductory talk in which participants expressed their general expectations and goals regarding the intervention program. After this short introductory talk, participants were asked to complete a questionnaire (on demographics, PA level and psychosocial variables; see Additional file 1). Thereafter, anthropometric measures (e.g., blood pressure, BMI, fat percentage) were collected by the PA counselors.

The measurement sessions consisted of two sessions that were separated by 1 week (Fig. 1). In the first session of each measurement period (session one, three and five), participants filled out the questionnaire and PA counselors collected anthropometric measures. Based on the participants’ anthropometry, a PA monitor (SenseWear Armband (SWA)) was prepared and participants were instructed to wear the SWA during the following week. In the second session of each measurement period (session two, four and six), results on the SWA and physical health measures (anthropometry) were discussed and compared with general recommendations for the specific parameters. In case of post- (session four) and follow-up (session six) measurement sessions, PA and physical health measures were compared to participants’ results on pre- and post-test, respectively.

In line with previously conducted PA interventions, the face-to-face counseling sessions lasted up to 60 min [6, 26]. During the first face-to-face counseling session (session 2), an individually tailored PA plan was designed based on participants’ goal, preferences for type of activities and current level of PA. Individualized PA programs were offered to facilitate common lifestyle PAs to improve cardiovascular health such as walking, cycling, running and swimming. Participants were allowed to choose one or more of the offered PA programs.

In addition, participants were encouraged to select at least one action out of a predefined list of actions to reduce their sedentary time and increase PA in multiple contexts (including home, transportation, workplace and leisure time). Proposed actions to reduce sedentary time included statements such as ‘I will stand during phone calls’ (work context) and ‘I will try to limit television viewing time’ (home context). The suggested actions to increase PA included statements such as ‘I will get off the bus one stop earlier’ (transportation context) and ‘I will take the stairs instead of the elevator’ (work/leisure time context). This opportunity to choose was created to support feelings of autonomy by enabling participants to individually select actions that they considered as relevant for themselves [37].

The three counseling contact moments between pre- and post-test were standardized and completed by e-mail or telephone depending on participant’s preference. The majority of participants (77%) chose to be contacted by e-mail, while nearly a quarter of participants (23%) preferred to be contacted by telephone. The content of the e-mail or telephone conversations was guided by the individualized PA plan and based on information on participant’s (baseline) PA behavior. More specifically, PA goals were evaluated and adjusted if necessary and participants were motivated to persist in and sustain their PA.

PA counseling was explicitly focused on fostering the three basic psychological needs outlined by SDT, (i.e., the need for autonomy, competence and relatedness) [16].

PA counselors intended to support *the need for autonomy* by allowing participants to choose from a number of options to facilitate lifestyle PAs (i.e., different types of individualized PA programs: walking, cycling, running and swimming programs). In addition, the need for autonomy was supported by providing participants with informational feedback (e.g., provide participants with individualized and personally relevant information regarding their PA preference) and by minimizing pressure (e.g., focus on facilitating rather than prescribing PA) during counseling sessions. These techniques encouraged participants to make informed decisions about the direction in which they preferred to proceed for the remainder of the intervention.

In order to support participants’ *need for competence*, PA counselors encouraged participants to consider how their intention(s) to become physically active might be implemented (i.e., implementation planning specified by the guiding questions ‘What, where, when, with whom will I be physically active?’; ‘What do I want to achieve?’; ‘How do I remind myself to be active?’). Implementing intentions based on these questions has been shown to be effective in previous studies [27, 38].

PA counselors intended to satisfy the *need for relatedness* by being empathetic (e.g., by demonstrating understanding), by providing positive feedback and by active listening during the counseling sessions. Moreover, participants were encouraged to engage in PA together with colleagues (e.g., form a group with colleagues to walk during lunch breaks) and seek support from colleagues. These techniques collectively aimed to stimulate participants to feel connected to both the PA counselor and colleagues alike.

Besides the need-supportive counseling, our intervention consisted of two additional behavior change techniques, namely barrier identification and self-monitoring [39]. Participants were asked to identify barriers (e.g., environmental or social) that could potentially deter them to engage in regular PA. They were asked to formulate ways to overcome the self-formulated potential barriers. With respect to self-monitoring, participants received pedometers (Omron, Walking Style One 2.1) and PA diaries offering participants an opportunity to goal set and self-monitor their PA behavior [40, 41].

The main goal of the intervention program was to increase participants’ baseline PA level.

Ideally, participants would adopt more PA into their daily lives and more participants would attain the recommended PA norm of 30 min of daily MVPA [4].

In the second face-to-face counseling session (session four), 14 weeks after the start of the intervention, participants’ PA behavior change was evaluated and future challenges for PA were discussed in order to encourage participants’ maintenance in PA engagement.

Between post- and follow-up sessions, no contact occurred between intervention group participants and PA counselors.

During the final session at follow-up (session six), participants were provided with an information leaflet. This information leaflet centered on future maintenance of PA and included tips to remain physically active after the intervention has ended.

##### Reference group

Participants in the reference group completed single pre-, post- and follow-up measurement sessions without receiving individualized PA counseling or a tailored PA plan. In the follow-up session (session three), the results with respect to their actual PA level were discussed and they were informed on the general PA recommendations.

#### Measures

Outcome measures were assessed at baseline, at post-intervention (immediately after the intervention) and at follow-up (6 months post-intervention; 9 months after baseline) (Fig. 1).

#### PA and sedentary behavior

PA behavior was objectively assessed by activity monitoring and assessed by self-report.

##### Objectively assessed PA and sedentary time

PA behavior was monitored by the SenseWear Pro3 Armband (BodyMedia, Inc. Pittsburgh, PA, USA) worn over the triceps muscle of the right arm. The SWA measures PA behavior using multiple sensors (i.e., tri-axial accelerometer, heat flux, skin temperature and galvanic skin response sensor) and was found to provide accurate, reliable and valid measures of PA [42–44]. Participants were instructed to wear the SWA for seven consecutive days (five weekdays and two weekend days) in order to provide reliable measures [45]. SWA data were combined with participants’ gender, age, height and body weight and were analyzed using computer-based SWA software (SenseWear professional software, version 7.0). Valid data included SWA data monitored on at least three weekdays and one weekend day for at least 720 min per day. PA intensity and daily step count were calculated. PA intensity was determined using MET values. Activities with a MET-value ≤1.8 were considered as sedentary [46]. Activities of light, moderate and vigorous intensity were defined as activities with a MET-value >1.8 and <3, ≥3 and <6, and ≥6, respectively [4]. In addition, time spent in combined MVPA was calculated by combining moderate PA with vigorous PA, respectively (moderate PA: vigorous PA = factor 1:2). Minute by minute activities were averaged into daily totals (min/day). To allow comparisons with the International Physical Activity Questionnaire (IPAQ) measures, modified ten-min bouts of accumulated activities were introduced. Bouts were defined as ten or more consecutive minutes of activity, allowing for interruptions of 1 or 2 min below the MET threshold [47, 48]. Activities were averaged on weekdays and weekend days separately. Weekdays and weekend days were also combined into an average day using the following formula: average day = (weekday average*5) + (weekend day average*2)/7.

Besides the daily minutes of the different intensities of PA, we also integrated the daily minutes of MVPA and number of daily steps into variables that represent the health-enhancing MVPA and steps guidelines. More specifically, we determined the number of participants meeting the recommended number of 10,000 steps per day (for adults) [49]. Furthermore, the number of participants achieving the recommended amount of 30 min of MVPA was determined [4]. The guideline measures were determined for weekdays, weekend days and average days separately.

##### Self-reported PA and sitting time

The 7-item short version of the IPAQ was used to measure self-reported PA. Participants had to report on how many days per week and for how long they engaged in walking, moderate- and vigorous-intensity PA (in bouts of 10 min) in the past seven days. In addition, participants reported the average minutes they spent sitting in the preceding seven days [50]. The IPAQ short form was found to have acceptable test-retest reliability [50]. The minutes of moderate and vigorous PA were combined (moderate PA: vigorous PA = factor 1:2) to form an MVPA measure. This MVPA measure was translated to the number of participants meeting the recommended amount of 30 min of daily MVPA [4].

#### Psychological need satisfaction

Participants’ perceived satisfaction of basic psychological needs was assessed by the previously validated 12-item Basic Psychological Needs in Exercise Scale (BPNES) [51, 52]. Participants indicated, on a five-point Likert scale (ranging from ‘I don’t agree at all’ to ‘I completely agree’), the extent to which they agree with the statements on autonomy (four items; e.g., I feel that I have the opportunity to make choices with regard to the way I exercise), competence (four items; e.g., I feel I have made a lot of progress in relation to the goal I want to achieve) and relatedness (three items; e.g., My relationships with the people I exercise with are close). The autonomy, competence and relatedness subscales were found to be internally reliable at pre, post and follow-up (Cronbach’s alpha ranged from .73 to .93).

#### Degree of autonomy support

To evaluate the need-supportive character of the intervention, we also examined participants’ perceptions of autonomy support provided by the PA counselors. At post-intervention, participants of the intervention group were questioned on the degree of perceived autonomy support using the short (six-item) version of the Health Care Climate Questionnaire (HCCQ) (e.g., exemplary item: I feel that my physical activity counselor provides me with choices and options) [53]. Intervention group participants indicated their agreement with the items on a seven-point Likert scale, ranging from totally disagree (=1) to totally agree (=7). The HCCQ items were marked by a high internal consistency (Cronbach’s alpha = .93).

#### Motivational regulations

Participants’ baseline level of motivation towards PA was compared using the Behavioural Regulation in Exercise Questionnaire-2 (BREQ-2) [54]. Participants indicated on a five-point Likert scale (ranging from ‘not true for me’ to ‘very true for me’) to what extent they had extrinsic (e.g., ‘I exercise because other people say I should’), introjected (e.g., ‘I feel guilty when I don’t exercise’), identified (e.g., ‘I value the benefits of exercise’) and intrinsic (e.g., ‘I exercise because it is fun’) reasons for participation in PA. A fifth subscale included amotivation (e.g., ‘I don’t see why I should have to exercise’). Following the example of previous studies, we combined subscales into the composite variables autonomous motivation (intrinsic motivation and identified regulation) and controlled motivation (introjected regulation and external regulation) [55–57]. Cronbach’s alpha ranged from .62 to .90 indicating an acceptable internal consistency.

### Statistical analyses

Data were analyzed using IBM SPSS (version 20; SPSS Inc., Chicago, IL, USA) and reported as mean and standard deviation in case of descriptives and as mean and standard error in case of outcome measures. Statistical significance was set at *p* < .05. Baseline differences between the intervention and reference group were examined by performing independent samples t-tests (for continuous measures) and chi-square tests (for categorical measures). Individual (at random) missing data on the BNPES questionnaire were imputed using the Expectation-Maximization procedure [58].

#### Outcome analyses

Outliers, defined as values exceeding three standard deviations from the mean, were identified in each outcome measure and excluded from analysis. Given the main advantage of mixed model analysis in handling with missing values, our longitudinal design allowed us to reliably assess the effects of the intervention with mixed model analysis [59]. Linear mixed model analysis with an unstructured covariance structure was used to determine time and intervention effects of the PA counseling intervention on objectively assessed PA, self-reported PA and psychosocial variables. To distinguish between short- and long-term intervention effects, two separate mixed model analyses were performed for each outcome variable. Two time points (pre and post) were used to determine short-term intervention effects and three time points (pre, post and follow-up) were used to examine long-term intervention effects.

For categorical variables (% of participants meeting PA guidelines), generalized estimating equations with an unstructured covariance structure were conducted to examine the short-term and long-term intervention effects. In case of between-group baseline differences, the baseline value was included as covariate in the mixed model analyses.

Cohen’s d effect sizes (ESs) were computed based on F or *χ* ^2^ statistics and sample sizes of the intervention and reference group [60]. ESs of < .30, .30-.80 and > .80 were considered small, medium and large effects, respectively [61].

#### Mediation analyses

Indirect effects of the PA counseling intervention (independent variable) on PA behavior (dependent variable) through perceived need satisfaction (mediators) were tested by the bootstrapping procedure using the SPSS PROCESS macro [62, 63]. Bias-corrected and accelerated confidence intervals (95% CI) of the indirect effects were generated with two thousand resamples. Bootstrapped CIs are preferred, as they make no unrealistic assumptions on the shape of the sampling distribution of indirect effects. Mediation analyses were conducted for change scores from pre- to post-test (short-term) and from pre- to follow-up-tests (long-term). Mediation was only tested in PA outcome measures which produced both short- and long-term intervention effects.

## Results

### Recruitment and follow-up

**Fig. 2:**
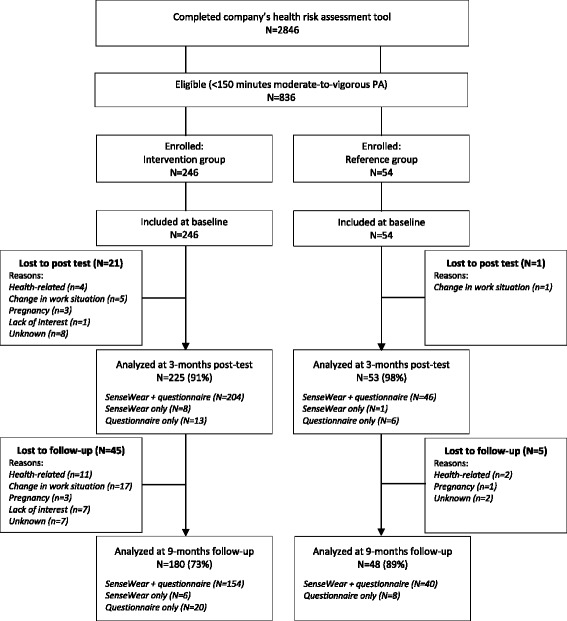
Participant flow chart

Of the 3264 people (45% female) employed at the two worksites, 2846 individuals (87%) completed the company’s HRA tool. From the 2846 respondents, 836 respondents (29%) scored less than the recommended PA level according to the HRA tool, whereof 246 individuals (29%) were included in the intervention group of the present study. Overall follow-up rate was 93% at 3-month post-test and 76% at 9-month follow-up. There was no difference in dropout rate at post-test (*χ* ^2^ = 2.912; *p* = .088). However, dropout rates differed at follow-up, with 27% dropout in the intervention group and 11% dropout in the reference group (*χ* ^2^ = 5.746; *p* = .017). Dropouts and non-dropouts did not differ in demographic variables, psychosocial variables, objectively assessed and self-reported PA, except for BMI. Dropouts were found to have a higher BMI than non-dropouts (*F* = 0.233; *p* = .017). Dropout rates and reasons for dropout are shown in Fig. 2.

### Participant characteristics

Table 1 provides baseline characteristics of the intervention and reference group. The majority of the included participants were female, were higher educated, lived with their partner and were overweight as indicated by a mean BMI ≥ 25 kg/m^2^. No differences were found between the intervention and reference group with respect to demographic variables, BMI, psychological need satisfaction and PA motivation. With respect to PA variables, significant baseline differences were observed in objectively assessed weekday sedentary time and weekday MVPA. More specifically, the intervention group demonstrated higher sedentary time (*F* = 4.495; *p* = .047) and lower MVPA during weekdays (*F* = 5.761; *p* = .042) compared to the reference group. Furthermore, participants in the intervention group reported higher weekday sitting time (*F* = 0.003; *p* = .038) and less walking (*F* = 3.187; *p* = .043) than participants in the reference group.

**Table 1:** Baseline characteristics of the intervention and reference group

	Intervention group (N = 246)	Reference group (N = 54)	F or *χ* ^2^ value
Age, mean (SD)	41.0 (8.8)	42.8 (8.5)	0.810
Gender, % female	76	80	0.257
BMI (kg/m^2^), mean (SD)	26.5 (4.4)	26.0 (4.4)	0.295
Living situation, % living together	91	96	4.041
Education, % higher/university education	88	84	1.112
SWA-based PA, mean (SD)			
Steps (n/day)			
Weekdays	9265.0 (3194.6)	10006.2 (3766.2)	3128
Weekend days	10092.3 (4181.5)	9597.3 (4506.6)	1.206
Average days	9501.4 (3088.2)	9889.4 (3478.8)	0.988
Sedentary time (min/day) (≤1.8 MET)			
Weekdays	1049.5 (130.7)	1005.2 (172.9)	4.495*
Weekend days	1005.8 (147.7)	998.8 (180.5)	1.758
Average days	1036.5 (118.4)	1003.4 (154.6)	3.385
Light PA (min/day) (>1.8- < 3 MET)			
Weekdays	63.7 (40.9)	72.5 (49.8)	3.998
Weekend days	93.6 (62.9)	99.8 (63.1)	0.000
Average days	72.3 (42.2)	81.8 (49.4)	1.797
Moderate PA (min/day) (>3- < 6 MET)			
Weekdays	34.0 (29.0)	44.1 (46.8)	6.493
Weekend days	52.0 (49.6)	51.2 (66.3)	2.587
Average days	40.6 (33.5)	46.6 (51.1)	4.757
Vigorous PA (min/day) (≥6 MET)			
Weekdays	1.7 (3.8)	3.1 (6.9)	16.025
Weekend days	1.4 (4.2)	0.7 (3.6)	3.358
Average days	1.6 (3.4)	2.4 (5.2)	7.422
MVPA (min/day) (≥3 MET)			
Weekdays	39.2 (34.4)	51.6 (50.8)	5.761*
Weekend days	57.8 (56.6)	52.7 (67.5)	0.774
Average days	44.6 (36.3)	52.4 (53.2)	5.936
Self-reported PA, mean (SD)			
Sitting week (min/day)	561.0 (161.0)	508.1 (151.7)	0.003*
Sitting weekend (min/day)	347.4 (159.7)	300.8 (134.6)	3.105
Walking (min/day)	106.1 (145.2)	155.4 (180.7)	3.187*
Moderate PA (min/day)	42.6 (66.8)	47.1 (67.5)	0.353
Vigorous PA (min/day)	25.2 (43.8)	31.2 (42.9)	0.361
Total PA (min/day)	191.7 (220.1)	237.0 (205.3)	0.101
Psychological need satisfaction, mean (SD)			
Autonomy	3.1 (0.8)	3.2 (0.9)	1.916
Competence	2.8 (0.7)	2.8 (0.8)	1.860
Relatedness	3.0 (1.1)	3.1 (1.0)	0.158
Total needs^a^	3.0 (0.7)	3.0 (0.7)	0.735
Exercise-related motivation, mean (SD)			
Amotivation	4.6 (0.5)	4.6 (0.5)	0.144
Controlled motivation^b^	2.4 (0.6)	2.3 (0.6)	0.436
Autonomous motivation^c^	3.9 (0.6)	4.0 (0.6)	0.377

*SWA* SenseWear Armband, *PA* Physical activity, *MVPA* Moderate-to-vigorous physical activity, *SD* standard deviation

*p < .05

^a^composite variable of autonomy, competence and relatedness need satisfaction; ^b^ composite variable of external and introjected regulation; ^c^composite variable of identified regulation and intrinsic motivation

### Intervention effects on PA and sedentary time

Tables 2 and 3 present the PA mean estimates (at pre, post and follow-up) and the short- and long-term intervention effects on objectively assessed and self-reported PA.

**Table 2:** Short- and long-term intervention effects on objectively assessed physical activity and sedentary time

	Intervention group			Reference group			Short-term intervention effect			Long-term intervention effect		
	Baseline	Post (3 months)	Follow-up (9 months)	Baseline	Post (3 months)	Follow-up (9 months)	Main effect of time	2 x 2 interaction^a^	ES	Main effect of time	3 x 2 interaction^b^	ES
	Mean (SE)	Mean (SE)	Mean (SE)	Mean (SE)	Mean (SE)	Mean (SE)	F	F	d	F	F	d
Steps (n/day)												
Weekdays	9173.1 (203.0)	10453.9 (207.7)	9646.4 (226.5)	10048.0 (446.7)	9521.2 (458.7)	10052.0 (460.1)	2.938	16.403***	0.65	1.530	8.988***	0.48
Weekend days	9851.5 (247.9)	11604.1 (290.9)	10971.9 (325.2)	9650.3 (548.2)	10418.0 (644.5)	9977.6 (646.8)	12.188**	1.730	0.21	6.744**	1.229	0.18
Average days	9378.6 (188.7)	10824.5 (197.7)	10034.7 (223.3)	9857.3 (416.4)	9767.8 (436.2)	9983.7 (456.4)	12.674***	13.966***	0.60	6.187	7.830**	0.45
Sedentary time (≤1.8 MET)												
Weekdays^c^	1039.1 (0.0)	994.5 (9.2)	1012.5 (9.6)	1039.1 (0.0)	1008.9 (20.5)	1044.4 (20.4)	13.015***	0.389	0.10	5.672	1.002	0.16
Weekend days	1005.2 (10.0)	949.3 (11.7)	976.1 (11.7)	997.5 (22.0)	969.4 (25.7)	987.0 (23.5)	9.094**	0.936	0.15	4.681	0.588	0.12
Average days	1035.1 (8.1)	986.4 (8.6)	1010.2 (9.5)	1001.3 (17.7)	969.7 (18.7)	1001.1 (19.3)	20.734***	0.972	0.16	10.345***	1.115	0.17
Light PA (>1.8- < 3 MET)												
Weekdays	64.1 (2.8)	69.5 (2.9)	68.0 (3.0)	74.2 (6.1)	73.7 (6.3)	70.9 (6.1)	0.217	1.506	0.20	0.372	0.744	0.14
Weekend days	94.5 (4.1)	93.7 (4.3)	102.9 (4.5)	98.5 (9.1)	89.1 (9.5)	93.9 (9.1)	0.954	0.685	0.13	0.781	0.819	0.14
Average days	73.1 (2.9)	77.2 (3.0)	78.4 (3.0)	82.6 (6.2)	77.8 (6.5)	77.3 (6.1)	0.116	2.734	0.26	0.009	1.673	0.21
Moderate PA (>3- < 6 MET)												
Weekdays	34.6 (2.2)	50.5 (2.8)	41.8 (2.9)	47.4 (4.8)	52.4 (6.1)	51.4 (5.8)	9.513**	2.733	0.26	5.322**	1.389	0.19
Weekend days	52.5 (3.5)	69.9 (4.6)	59.6 (4.5)	55.1 (7.7)	71.5 (10.1)	64.6 (9.1)	8.932**	0.049	0.04	5.063**	0.048	0.04
Average days	41.3 (2.5)	57.3 (3.0)	47.6 (3.0)	51.3 (5.5)	58.5 (6.6)	54.8 (6.1)	12.020**	2.083	0.23	6.577**	0.950	0.16
Vigorous PA (≥6 MET)												
Weekdays	1.7 (0.3)	3.5 (0.4)	3.2 (0.5)	3.0 (0.6)	2.5 (0.9)	4.1 (0.9)	1.791	4.704*	0.35	2.889	2.625	0.26
Weekend days	1.4 (0.3)	4.5 (0.6)	4.0 (0.7)	0.7 (0.6)	2.8 (1.3)	5.0 (1.4)	12.733***	0.406	0.10	14.044***	1.095	0.17
Average days	1.6 (0.2)	3.8 (0.4)	3.3 (0.4)	2.4 (0.6)	2.6 (0.9)	4.4 (0.9)	6.928**	3.008	0.28	8.285***	2.696	0.26
MVPA (≥3 MET)												
Weekdays^d^	41.3 (0.0)	60.3 (3.4)	51.1 (3.1)	41.3 (0.0)	42.5 (7.6)	43.3 (6.7)	5.905*	4.582*	0.34	3.052	2.281	0.24
Weekend days	57.7 (3.8)	81.2 (5.1)	69.3 (5.0)	56.1 (8.5)	78.1 (11.1)	79.1 (10.1)	13.201***	0.063	0.04	8.870***	0.712	0.14
Average days	45.2 (2.6)	65.3 (3.3)	54.8 (3.2)	56.4 (5.8)	65.4 (7.2)	64.3 (6.5)	14.478***	2.316	0.24	8.190***	1.263	0.18

PA and sedentary time are expressed as minutes per day

*PA* Physical activity, *MVPA* Moderate-to-vigorous physical activity, *MET* Metabolic equivalent, *ES* Effect size, *SE* Standard error

*p < .05, **p < .01, ***p < .001

^a^2(time) x 2(group) interaction effect; ^b^3(time) x 2(group) interaction effect; ^c^ adjusted for baseline weekday sedentary time; ^d^adjusted for baseline weekday MVPA

**Table 3:** Short- and long-term intervention effects on self-reported physical activity and sitting time

	Intervention group			Reference group			Short-term intervention effect			Long-term intervention effect		
	Baseline	Post (3 months)	Follow-up (9 months)	Baseline	Post (3 months)	Follow-up (9 months)	Main effect of time	2x2 interaction^a^	ES	Main effect of time	3x2 interaction^b^	ES
	Mean (SE)	Mean (SE)	Mean (SE)	Mean (SE)	Mean (SE)	Mean (SE)	F	F	d	F	F	d
Sitting week^c^	551.1 (0.0)	477.9 (11.6)	508.0 (12.4)	551.1 (0.0)	513.9 (25.9)	504.5 (12.4)	15.327***	1.549	0.19	8.461***	1.251	0.17
Sitting weekend	349.7 (10.1)	305.4 (10.9)	340.2 (12.6)	299.7 (22.0)	330.3 (22.1)	316.3 (24.8)	0.286	8.131**	0.44	0.423	4.469*	0.32
Walking^d^	111.2 (0.0)	127.6 (10.2)	97.3 (11.3)	111.2 (0.0)	77.7 (22.7)	50.5 (24.3)	0.349	3.540	0.29	4.776**	2.205	0.23
Moderate PA	43.2 (4.3)	80.7 (5.8)	58.9 (5.5)	47.5 (9.4)	64.1 (12.0)	48.3 (10.5)	13.790***	2.020	0.22	6.940**	1.130	0.16
Vigorous PA	25.8 (2.8)	69.5 (5.8)	46.1 (4.7)	30.8 (6.1)	55.7 (12.0)	55.3 (9.0)	26.983***	2.056	0.22	17.384***	1.430	0.18
Total PA	194.8 (14.1)	280.6 (12.7)	205.9 (11.1)	242.3 (31.5)	254.9 (26.5)	207.6 (21.4)	7.873**	3.729	0.29	7.522**	1.943	0.21

PA and sitting time are expressed as minutes per day

*PA* Physical activity, *SE* Standard error, *ES* Effect size

*p < .05, **p < .01, ***p < .001

^a^2(time) x 2(group) interaction effect; ^b^3(time) x 2(group) interaction effect; ^c^adjusted for baseline weekday sitting; ^d^adjusted for baseline walking

#### Objectively assessed PA and sedentary time

##### Short-term intervention effects

In the short-term, participants in the intervention group increased their number of steps during weekdays (+1281 steps/day), while reference group participants accumulated fewer steps than at baseline (-527 steps/day) (*p* < .001; ES = .65). A similar pattern was observed in daily step count during week and weekend days combined. Intervention group participants increased their daily steps by 1446 steps per day, while participants in the reference group decreased their steps by 89 steps per day (*p* < .001; ES = .60).

Furthermore, a significant short-term intervention effect was observed in participants’ weekday vigorous PA and MVPA. The intervention group increased their daily minutes of vigorous PA by 2.3 min/day, while a decrease of 0.5 min/day was observed in the reference group (*p* = .031; ES = .35). Daily minutes of MVPA increased more distinctly in the intervention group by 17.8 min/day compared to the reference group (*p* = .033; ES = .34). No short-term intervention effects were found for sedentary time, light PA and moderate PA.

##### Long-term intervention effects

The significant short-term intervention effects on accumulated number of daily steps during weekdays, and on week and weekend days combined were sustained in the long-term. During weekdays, the intervention group more distinctly increased daily step count by 469 steps/day compared to the reference group (*p* < .001; ES = .48). Daily step count during week and weekend days combined showed the same pattern. Participants in the intervention group demonstrated a more pronounced increase in daily step count of 530 steps/day compared to participants in the reference group participants (*p* = .001; ES = .45).

There were no significant long-term intervention effects on sedentary time, light PA, moderate PA and MVPA.

#### Self-reported PA and sitting time

##### Short-term intervention effects

Following the intervention, self-reported sitting time during weekend days was significantly reduced in the intervention group (-44 min/day) while the reference group had increased their level of sitting (+31 min/day) (*p* = .005; ES = .44). Weekday sitting, moderate PA and vigorous PA did not show short-term intervention effects.

##### Long-term intervention effects

The short-term intervention effect on self-reported weekend day sitting time was sustained in the long-term. At follow-up, participants in the intervention group reported lower levels of sitting during the weekend (-10 min/day) whereas the reference group reported more sitting time during weekend days (+17 min/day) (*p* = .012; ES = .32). No long-term intervention effects were found for weekday sitting, walking, moderate PA, vigorous PA and total PA.

#### PA guidelines based on objectively assessed and self-reported PA

Table 4 presents the percentages of participants meeting steps and MVPA guidelines (at pre, post and follow-up) and the short- and long-term intervention effects on participants meeting these guidelines.

**Table 4:** Short- and long-term intervention effects on participants meeting objectively assessed and self-reported MVPA and steps guidelines

	Intervention group			Reference group			Short-term intervention effect			Long-term intervention effect		
	Baseline	Post (3 months)	Follow-up (9 months)	Baseline	Post (3 months)	Follow-up (9 months)	Main effect of time	2x2 interaction^a^	ES	Main effect of time	3x2 interaction^b^	ES
	%	%	%	%	%	%	*χ* ^2^	*χ* ^2^	d	*χ* ^2^	*χ* ^2^	d
SWA-based PA												
Steps guideline^c^, % meeting guideline												
Weekdays	34	51	43	40	34	44	1.997	9.548**	0.37	3.658	10.107**	0.38
Weekend days	43	61	56	42	46	51	5.357*	1.580	0.15	6.071*	1.953	0.16
Average days	36	58	46	38	35	44	7.545**	12.422***	0.42	8.041*	13.910**	0.45
MVPA guideline^d^, % meeting guideline												
Weekdays	50	67	66	60	62	63	4.949*	2.804	0.20	5.617	2.787	0.20
Weekend days	63	72	69	43	62	58	8.751**	0.883	0.11	10.034**	1.048	0.12
Average days	55	76	71	53	69	63	21.261***	0.537	0.09	21.846***	0.721	0.10
Self-reported PA												
MVPA guideline^d^, % meeting guideline	61	83	76	63	66	81	10.167**	6.163*	0.29	15.406***	9.408**	0.36

*SWA* SenseWear Armband, *PA* physical activity, *MVPA* moderate-to-vigorous physical activity, *ES* Effect size

*p < .05, **p < .01, ***p < .001

^a^2(time) x 2(group) interaction effect; ^b^3(time) x 2(group) interaction effect; ^c^ ≥ 10,000 steps per day; ^d^ ≥ 30 min of daily moderate-to-vigorous physical activity

##### Short-term intervention effects

Based on objective assessments, the percentage of participants who met the recommended steps guideline (i.e., 10,000 steps per day) [49] showed a short-term intervention effect. During weekdays, the percentage of participants in the intervention group meeting the steps guideline increased from 34% at baseline to 51% at post-intervention (+17%). Meanwhile, less reference group participants met the steps guideline after the intervention (-6%) (*p* = .002; ES = .37). A similar pattern was observed in participants meeting the steps guideline based on week and weekend days combined. More intervention group participants met the steps guideline after the intervention (+22%), while slightly less reference group participants met the steps guideline at post-intervention (-3%) (*p* < 0.001; ES = .42).

We found mixed results with regards to participants meeting the recommended MVPA guideline (30 min of daily moderate-to-vigorous PA). Based on self-report, more participants met the MVPA guideline in the intervention group (+22%) relative to the reference group (+3%) (*p* = .013; ES = .29). Meanwhile, no short-term intervention effects were found on participants meeting the MVPA guideline according to objective assessments.

##### Long-term intervention effects

The short-term intervention effects on participants meeting the steps guideline were sustained in favor on the intervention group. The percentage of participants meeting the steps guideline showed a more pronounced increase among intervention group participants (+9% and +10% on weekdays and week and weekend days combined, respectively) compared to reference group participants (+4% and +6% on weekdays and week and weekend days combined, respectively) (*p* = .006; ES = .38 for weekdays and *p* = .001; ES = .45 for week and weekend days combined). In addition, the short-term intervention effects on participants meeting the MVPA guideline according to self-reported MVPA were maintained in the long-term (*p* = .009; ES = .36). With respect to participants meeting the objectively derived MVPA guideline, no long-term intervention effects were found.

### Intervention effects on psychological need satisfaction

The mean estimates (at pre, post and follow-up) and short- and long-term intervention effects on psychological need satisfaction are presented in Table 5.

**Table 5:** Short- and long-term intervention effects on psychological need satisfaction

	Intervention group			Reference group			Short-term intervention effect			Long-term intervention effect		
	Baseline	Post (3 months)	Follow-up (9 months)	Baseline	Post (3 months)	Follow-up (9 months)	Main effect of time	2x2 interaction^a^	ES	Main effect of time	3x2 interaction^b^	ES
	Mean (SE)	Mean (SE)	Mean (SE)	Mean (SE)	Mean (SE)	Mean (SE)	F	F	d	F	F	d
Need satisfaction (1–5)												
Autonomy	3.05 (0.05)	3.51 (0.05)	3.43 (0.06)	3.17 (0.11)	3.32 (0.10)	3.30 (0.12)	21.517***	5.331*	0.35	10.860***	3.002	0.26
Competence	2.80 (0.05)	3.17 (0.05)	2.96 (0.07)	2.77 (0.10)	2.77 (0.11)	2.72 (0.13)	6.820**	6.803*	0.40	3.889*	3.894*	0.30
Relatedness	3.03 (0.07)	3.14 (0.07)	2.92 (0.09)	3.05 (0.15)	3.06 (0.14)	2.92 (0.18)	0.538	0.332	0.09	1.806	0.227	0.07
Total needs^c^	2.96 (0.04)	3.27 (0.04)	3.11 (0.05)	3.00 (0.09)	3.05 (0.09)	3.00 (0.11)	10.260**	5.085*	0.34	5.664**	3.060*	0.27

*SE* Standard error, *ES* Effect size

*p < .05, **p < .01, ***p < .001

^a^2(time) x 2(group) interaction effect; ^b^3(time) x 2(group) interaction effect; ^c^composite variable of autonomy, competence and relatedness need satisfaction

#### Short-term intervention effects

Perceived autonomy, competence and total need satisfaction increased significantly higher among intervention group participants by, respectively, 0.31, 0.37 and 0.26 compared to reference group participants (*p* = .022; ES = .35, *p* = .010; ES = .40, *p* = .025; ES = .34 for autonomy, competence and total need satisfaction, respectively). No short-term intervention effects were observed for satisfaction of perceived relatedness.

#### Long-term intervention effects

There were sustained intervention effects on competence and total need satisfaction in the long-term. Between-group change differences of 0.18 and 0.15 were observed in competence (*p* = .022; ES = .30) and total need satisfaction (*p* = .049; ES = .27), respectively. Satisfaction of perceived autonomy and perceived relatedness did not show long-term intervention effects.

### Mediation effects of psychological need satisfaction on daily step count

Objectively assessed daily step count during weekdays and week and weekend days demonstrated combined short- and long-term intervention effects. Consequently, mediation analysis was examined in these two outcome variables. The mediating (indirect) effects of the need satisfaction subscales on daily step count are shown in Table 6.

**Table 6:** Mediating effects of psychological need satisfaction on changes in objectively assessed daily step count

	Short-term (3 months)			Long-term (9 months)		
	α-path	β-path	αβ-path	α-path	β-path	αβ-path
Mediator variables	B (SE)	B (SE)	B (95% CI)	B (SE)	B (SE)	B (95% CI)
Need satisfaction						
Autonomy						
Weekday steps	0.24 (0.15)	620.97 (196.74)**	151.57 (-0.47–421.36)	0.37 (0.16)*	410.34 (221.57)	151.43 (13.64–419.73)*
Average day steps	0.23 (0.15)	436.83 (176.81)*	100.83 (-3.33–76.86)	0.36 (0.16)*	538.01 (195.02)**	195.68 (29.42–510.41)*
Competence						
Weekday steps	0.40 (0.17)*	626.11 (168.59)***	249.93 (76.80–541.99)*	0.34 (0.18)	514.84 (198.98)*	173.22 (20.86–454.86)*
Average day steps	0.39 (0.17)*	498.37 (151.38)**	196.23 (50.97–433.38)*	0.34 (0.18)	576.19 (176.03)**	194.44 (25.47–447.12)*
Relatedness						
Weekday steps	0.06 (0.19)	517.67 (151.04)***	28.85 (-145.65–240.08)	0.12 (0.21)	-63.58 (172.17)	-7.53 (-137.01–38.26)
Average day steps	0.04 (0.19)	365.89 (136.62)	13.92 (-104.44–166.38)	0.08 (0.22)	88.76 (150.16)	7.08 (-33.93–134.05)
Total needs^a^						
Weekday steps	0.23 (0.13)	942.42 (212.80)***	219.59 (3.51–508.61)*	0.27 (0.14)*	431.44 (261.95)	118.50 (9.61–366.94)*
Average day steps	0.22 (0.14)	695.09 (192.17)***	153.53 (0.65–394.47)*	0.26 (0.14)	646.93 (230.33)**	168.41 (15.44–431.02)*

α, estimate of the intervention effect on changes in the proposed mediators; β, estimate of the direct effect of changes in the proposed mediators on changes in daily step count while controlling for the intervention effect; αβ, estimate of the indirect effect of the intervention on changes in daily step count through the proposed mediators; 95% CI, 95% bias-corrected and accelerated confidence interval based on 2000 bootstrap resamples

*B* Unstandardized regression coefficient, *SE* Standard error

*p < .05, **p < .01, ***p < .001

^a^composite variable of autonomy, competence and relatedness need satisfaction

#### Short-term mediation

Mediation analyses demonstrated significant effects of the intervention on short-term changes in perceived competence satisfaction (α-path), indicating that the intervention group increased more in competence satisfaction than the reference group (*p* = .021, *p* = .024 for weekday and average day steps, respectively).

Significant β-paths were observed in almost all mediator paths (except for relatedness satisfaction on average day steps). This means that, the more participants increased in need satisfaction, the more they increased their daily step count.

We also observed significant indirect αβ-pathways for competence satisfaction (B = 249.9; 95% CI: 76.8–542.0, B = 196.2; 95% CI: 51.0–433.4 for weekday and average day steps, respectively) and total need satisfaction (B = 219.6; 95% CI: 3.5–508.6, B = 153.5; 95% CI: 0.7–394.5 for weekday and average day steps, respectively). This indicates the mediating role of competence satisfaction and total need satisfaction in the short-term effects of the intervention on daily step count. Autonomy and relatedness satisfaction did not result in short-term mediating effects.

#### Long-term mediation

As indicated by the α-paths, significant long-term effects of the intervention were found on autonomy (*p* = .024, *p* = .028 for weekday and average day steps, respectively) and total need satisfaction (*p* = .046 for weekday steps). There were also significant direct effects of changes in autonomy, competence and total need satisfaction on long-term changes in daily step count (β-paths). This indicates that the long-term increases in autonomy, competence and total need satisfaction corresponded with long-term increases in daily step count.

Significant mediating effects were found for autonomy (B = 151.4; 95% CI: 13.6–419.7, B = 195.7; 95% CI: 29.4–510.4 for weekday and average day steps, respectively), competence (B = 173.2; 95% CI: 20.9–454.9, B = 194.4; 95% CI: 25.5–447.1 for weekday and average day steps, respectively) and total need satisfaction (B = 118.5; 95% CI: 9.6–366.9, B = 168.4; 95% CI: 15.4–431.0 for weekday and average day steps, respectively). As in the short-term, no long-term mediating effects of relatedness satisfaction were found.

### Degree of autonomy support

After the intervention, participants of the intervention group indicated a mean score of 6.03 ± 0.68 on a seven-?A3B2 twb=.3w?>point Likert scale. Subsequent analyses indicated a minimum score of 4 with 63.4% of participants scoring 6 or more.

## Discussion

The main objective of the present study was to evaluate the short- and long-term effects of an individualized need-supportive PA counseling intervention on employees’ PA and sedentary behavior. In line with our first hypothesis, the results of this study demonstrated that a 3-month individualized need-supportive PA counseling intervention was effective in increasing the number of weekday daily steps, both in the short- and long-term.

With regards to objectively assessed PA, we found short-term effects on weekday vigorous PA and MVPA. Meanwhile, no intervention effects were found on the remaining PA intensities. It should be noted however that main effects of time emerged on objectively assessed moderate PA, vigorous PA and MVPA, indicating an increase in PA levels in both the intervention and reference group. The fact that the participants in the reference group increased objectively assessed PAs could be the result of measurement reactivity [64]. Another explanation of the observed reference group improvements relates to the fact that there were overlapping PA initiatives at the worksites throughout the measurement periods. An elaborate discussion on the content of these overlapping PA initiatives can be found in the strengths and limitation section. The increased PA level among reference group participants might also be the result of the (objective) monitoring of their PA level (i.e., measurement reactivity). This increased PA among reference group participants is a common phenomenon observed in behavioral intervention trials, especially in interventions that exclude participants who meet the PA guidelines (as was the case in the current intervention) [65].

The fact that we found significant intervention effects in daily step count and not in the different PA intensities could be due to the use of pedometers among intervention group participants. Pedometers provide instant feedback on the number of accumulated steps, and this immediate feedback might have facilitated this specific movement pattern. This interpretation is supported by a recent intervention study, in which participants increased their PA levels (including number of steps) when they received pedometers combined with need-supportive coaching [66]. It is important to note, however, that providing pedometers alone did not increase PA levels throughout the intervention. Individualized need-supportive PA coaching was considered as crucial to increase and sustain PA levels [66].

In order to determine the societal relevance of the post-intervention increases in daily step count, we translated the variables into the health-enhancing steps guideline of 10,000 steps per day [49]. The number of participants who met the steps guideline increased considerably in the intervention group, both in the short- and long-term. In fact, more than half of intervention group participants met the steps guideline after the intervention (51% for weekdays and 58% for average days). In addition to the steps guideline, we also examined the number of participants meeting MVPA guideline of 30 min of daily MVPA [4]. The intervention resulted in an increase in the number of participants who self-reportedly met the MVPA guidelines, both in the short- and long-term. Objectively assessed MVPA guideline measures did not show intervention effects. The fact that we found intervention effects in self-reported MVPA and not in objective MVPA measures, could be the result of an overestimation of higher intensity PAs in self-reported measures compared to objective PA assessments [46].

At baseline, a subset of participants in the intervention group showed sufficient levels of PA based on both self-reported and objective measures of PA. The fact that some intervention group participants adopted sufficient PA levels was conflicting with the inclusion criteria, as we attempted to only include insufficiently active employees. The discrepancy between the inclusion measures and the baseline measures could be due to differences in questioning between the company’s HRA tool (used as inclusion criterion) and the IPAQ (used as self-reported PA measure). The HRA tool emphasized higher intensity activities, e.g., exercise, while the IPAQ also referred to activities of light intensity such as walking. Moreover, the HRA tool referred to the last thirty days whereas the IPAQ recalled on the last seven days. In addition, there could be a substantial time period (up to 3 months) between assessment of the HRA tool and administering of the IPAQ. Another factor that could have contributed to the discrepancy is the possibility that HRA triggered employees to become more physically active prior to baseline assessments. The limitations of self-reported PA screening tools are discussed in previous studies [67, 68]. In fact, it is not uncommon among PA intervention trials, that relatively active participants are recruited, even when the initial aim was to only include low-active participants. It has therefore been suggested to alternatively use objective PA assessments as screening tools, although these objective tools involve significant cost and time investments [67].

Despite the fact that the counseling was mainly focused on changing employees’ PA behavior, a short- and long-term reduction in self-reported sitting time during weekends was found. The specific reduction of self-reported sitting time in the weekends might result from the fact that most participants spend more time exercising and participating in sports on weekend days. This engagement in higher intensity PAs might, in part, have replaced sitting time [69, 70]. We found main effects of time in objectively assessed weekend days’ moderate, vigorous and MVPA indicating an increase of higher intensity PAs in both the intervention and reference group. Moreover, the reduction of sitting time was targeted as a secondary outcome in our study, which might explain the lack of intervention effects on weekday sitting time. Martin et al. indicated smaller intervention effects when reducing sedentary behavior was considered as a secondary outcome [71].

A recent comprehensive systematic review examined the influence of interventions on sedentary time in adults and distinguished between PA interventions, PA and sedentary behavior interventions and sedentary behavior interventions [72]. Our study could be categorized as a PA and sedentary behavior intervention, as we also included intervention components to reduce sedentary behavior (e.g., participants were offered list of options to reduce their sedentary time at the workplace and at home/leisure time such as standing up during phone calls). While the review reported an overall intervention effect of 35 min per day (of PA and sedentary behavior intervention) [72], we found comparable intervention effects of 75 (short-term) and 27 min per day (long-term). A more recent meta-analysis, including only RCTs, found comparable results, albeit of less magnitude [71]. Interventions at the workplace were found to result in a non-significant reduction in sedentary time of nearly 9 min per day [71].

The second objective was to evaluate mediating effects of psychosocial variables targeted by the PA counseling intervention. We evaluated the mediating influence of psychological need satisfaction as the PA counselors aimed to satisfy the participants’ basic psychological needs. The short-term intervention effects were mediated by changes in perceived competence need satisfaction and total need satisfaction, while the long-term intervention effects were mediated by changes in autonomy, competence and total need satisfaction. Previous evidence revealed a strong inter-correlation between the three needs (in particular between autonomy and competence) [73]. Therefore, the use of a (unidimensional) total needs variable seems to be justified [74]. As a consequence, the short- and long-term mediation effects of changes in total need satisfaction on changes in daily steps confirmed our expectations (hypothesis 2) and imply that the intervention resulted in significant intervention effects through satisfaction of the three needs, which is consistent with SDT-based assumptions [25]. There were, however, no mediating effects of relatedness satisfaction. These findings are in line with previous studies as a comprehensive review indicated no (cross-sectional) association between relatedness satisfaction and exercise/PA [25]. The authors of the review provided a possible explanation of this lack of association, by pointing out that engagement in solitary activities (such as walking or jogging) might be satisfying without a specific need for relatedness [25].

The autonomy supportive character of the counseling intervention was questioned among intervention group participants. The high mean scores (6.03; min-max: 4–7 on a 7-point Likert scale) indicated that participants in the intervention group reported a high degree of autonomy support from the PA counselors during the counseling sessions. The autonomy supportive climate throughout counseling sessions might have contributed to the observed intervention effects.

### Strengths and limitations

The strength of this study relates to the fact that it is among the first to evaluate the long-term effectiveness of an SDT-based worksite PA intervention among employees. The combination of subjective and objective assessments of PA forms the second strength of our study. A third strength concerns the examination of both (short- and long-term) intervention and mediation effects of the PA counseling intervention and psychosocial variables, respectively.

Nevertheless, our study also had some limitations that should be taken into account. A first limitation is the lack of full randomization. Because of the service providing nature of the PA counseling program to the company in which the intervention took place, we considered it to be unethical and inappropriate to fully randomize participants to the intervention or control condition. This had possible consequences on the extrapolation of our findings as a recent review on worksite PA interventions pointed out that randomized controlled trials were less likely to be effective compared to quasi-experimental studies [12]. The review indicated that the included worksite RCTs were more likely to be of longer duration than the controlled quasi-experiments. As speculated by the review’s authors, the longer duration (>6 months) of worksite RCTs might explain the diminished effectiveness of these RCTs as it is more difficult to sustain an intervention effect in the long-term [12].

A second limitation is the presence of overlapping worksite initiatives at the time of the intervention. There were overlapping initiatives related to promotion of PA and well-being including a *Start to Run* program and a well-being program called *Energy for Performance in Live* (EFPIL). These initiatives included sessions in which employees were informed about ‘the do’s and don’ts’ when intentions were made to *Start to Run*. Employees were also informed on how to keep their energy level up throughout their workday (EFPIL). These worksite initiatives might have influenced the study participants in terms of their PA level. However, participants were questioned on whether they participated in one of the overlapping worksite health promotion initiatives (EFPIL). Eighty-nine percent of intervention group participants and 92% of reference group participants reported that they did not participate in EFPIL. This suggests that the majority of participants made no use of this specific worksite initiative.

Besides the overlapping worksite initiatives, a third limitation refers to the risk of contamination across participants in the intervention and reference group, as we recruited both groups within the same worksites and during the same recruitment period. We cannot rule out the fact that participants in the intervention group have influenced their colleagues belonging to the reference group.

The difference in follow-up dropout rates constitutes a fourth limitation. At follow-up, there was a higher dropout in the intervention group (27%) compared to the reference group (11%). The higher dropout specific in the intervention group may be the result of a lack of contact moments between post and follow-up measurement sessions. This notion is strengthened given that there was no between-group difference in dropout rate at post-intervention (9% and 2% dropout in the intervention and reference group, respectively). Besides the difference in follow-up dropout rates, we also found that dropouts had a higher BMI than non-dropouts. This finding is not uncommon in lifestyle interventions [75, 76] and should be taken into consideration when targeting overweighed individuals. Accordingly, future PA behavioral interventions should develop and implement strategies to prevent insufficiently active, overweighed individuals from dropping out.

A fifth limitation relates to the fact that the study population consisted of predominantly female and highly educated participants. This self-selection may have compromised the generalizability of our findings to other workplaces.

## Conclusions

The findings of the current study indicate that a 3-month individualized need-supportive PA counseling intervention delivered at the workplace elicited short- and long-term intervention effects on daily step count, self-reported weekend day sitting and psychological need satisfaction. Besides the short- and long-term intervention effects on need satisfaction, autonomy and competence satisfaction were found to mediate the long-term intervention effectiveness.

The relative few contact sessions (two face-to-face counseling sessions and three contacts by e-mail or telephone) and small-to-moderate effect sizes suggest a potential cost-effective PA intervention [77]. Future research should consider the cost-effectiveness of a comparable intervention and evaluate this type of worksite intervention in a randomized controlled setting with longer follow-up. Overall, our findings contribute to the growing evidence that need-supportive PA counseling could be implemented as part of larger, community-based PA promotion programs.

## Additional file

Additional file 1: Questionnaire used during the measurement sessions (DOCX 50 kb)

## Abbreviations

BPNES: Basic psychological needs in exercise scale
BREQ-2: Behavioural regulations in exercise questionnaire-2
EFPIL: Energy for performance in live
HCCQ: Health care climate questionnaire
HRA: Health risk assessment
IPAQ: International Physical Activity Questionnaire
MVPA: Moderate-to-Vigorous Physical Activity
PA: Physical activity
PAR-Q: Physical Activity Readiness Questionnaire
SDT: Self-determination theory
SWA: SenseWear Armband
