# Supplementary material for: Short- and long-term effectiveness of a three-month individualized need-supportive physical activity counseling intervention at the workplace
Source: BMC Public Health. 2017 Jan 9;17:52. doi: 10.1186/s12889-016-3965-1 (PMC5223544; doi:10.1186/s12889-016-3965-1)
Supplement: Additional file 1: — Questionnaire used during the measurement sessions (DOCX 50 kb) [file 12889_2016_3965_MOESM1_ESM.docx]

**Additional file 1:** Questionnaire used during the measurement sessions

**PART I GENERAL & HEALTH**

| ***Question 1.**** | *Who are you? Please provide the following personal information.* | | | | |
| --- | --- | --- | --- | --- | --- |
| 1. Last name: | | ________________________ | |  |  |
| First name: | | ________________________ | |  |  |
| 1. Sex: | | - Male | | - Female |  |
| 1. Living situation: | | - Live alone (without partner nor kids or others) | | Live together  (multiple options possible):   - - With partner   - With other(s): (e.g. children, parents): _______________ |  |
| 1. Children | | - No | - Yes, age(s)? _____________________________ | |  |
| 1. Level of education | | - No diploma - Highschool | | - Higher education (no university) - University |  |

* only included in baseline questionnaire

| ***Question 2a.*** | *How is your physical health?*  *This questionnaire informs us about disorders and risk factors for cardiovascular diseases and will be used to offer you an appropriate physical activity program. Please indicate whether the following statements are true for you by checking No or Yes.* | | |
| --- | --- | --- | --- |
| 1. Has your doctor ever said that you have a **heart condition** and that you should only do physical activity recommended by a doctor? | | - No | - Yes |
| 1. Do you feel **pain in your** chest when you do **physical activity**? | | - No | - Yes |
| 1. In the past month, have you had **chest pain** when you were **not** **doing physical activity**? | | - No | - Yes |
| 1. Do you **lose your balance** because of dizziness or do you ever **lose consciousness**? | | - No | - Yes |
| 1. Do you have a **bone or joint problem** (for example, back, knee or hip) that could be made worse by a change in your physical activity? | | - No | - Yes |
| 1. Do you know of **any other reason** why you should not do physical activity? | | - No | - Yes |
| 1. Is your doctor currently prescribing **drugs** (for example, water pills) for your blood pressure or heart con­dition? | | - No | - Yes |

| ***Question 2b.*** | *For the following conditions/diseases, please put a cross under the columns that are true for your situation. Please answer honestly.* | | | |  |
| --- | --- | --- | --- | --- | --- |
| **Conditons/diseases** | | Has a doctor or other health worker ever told you that you suffer from ... | Are you currently suffering from …/taking medication for.... | In the past three days  did you take  medication for ... | |
| 1. ... high **blood pressure**? | |  |  |  | |
| 2. ... high **cholesterol**? | |  |  |  | |
| 1. ... **diabetes**? | |  |  |  | |
| 1. ... a **heart disease**?  (e.g. congenital heart disease, valvular heart disease, valve noise, ever had a heart attack, constricting chest pain (angina) that radiates to the arm or jaw) | |  |  |  | |
| 1. ... a disease of the **blood vessels**?  (e.g. blockage, narrowing of arteries, saccular expansions of blood vessels (aneurysm), stroke, ever had surgery on the arteries, blood clots, phlebitis) | |  |  |  | |
| 1. ... a **long disease**?  (e.g. chronic bronchitis, chronic pulmonary disease or COPD, asthma, lung cyst) | |  |  |  | |
| 1. ... a **muscle** or **joint** disease/ injury? If yes, what injury/disease? ____________________ | |  |  |  | |
| 1. … a **back** injury/ (lower) back pain? | |  |  |  | |
| 1. ... **sleeping disorder**? | |  |  |  | |
| 1. ... **stress/ anxiety**? | |  |  |  | |
| 1. … **depression**? | |  |  |  | |

| ***Question 2c.*** | *Please mark which of the following statements are true for you.* | |  |
| --- | --- | --- | --- |
| 1. Do you smoke? | | - I have never smoked. - I have smoked but I quit ... months/ ... years ago. - I smoke every day/ at least every week/ at least every month | |
| 1. Are you pregnant? | | - No - Yes | |

| ***Question 2d.*** | *Health and work* | | | | | | | | | | |
| --- | --- | --- | --- | --- | --- | --- | --- | --- | --- | --- | --- |
| 1. **How often** have you called in sick in the past 12 months ? 2. **How many days** in total couldn’t you work because of sickness in the past 12 months? | | | ____times  ____days | | | | | | | | |
| *How often have you called in sick in the past 12 months because of the following reasons?* | | NEVER | | SOMETIMES | | REGULARLY | | OFTEN | | VERY OFTEN | |
| 1. **Physical work related** reasons (rsi, back pain, etc.) | | 1 | | | 2 | | 3 | | 4 | | 5 |
| 1. **Psychological work related** reasons (workload, tensions at work, etc.) | | 1 | | | 2 | | 3 | | 4 | | 5 |
| 1. **Physical private** reasons (fever, common cold, etc.) | | 1 | | | 2 | | 3 | | 4 | | 5 |
| 1. **Psychological private** reasons (tensions at home, psychological problems, etc.) | | 1 | | | 2 | | 3 | | 4 | | 5 |

| ***Question 3.*** | *How do you feel?*  *Please indicate how frequently the following statements were true for you in the past month. In the scale below, please circle the number (from 1 to 7)* *that* ***best fits*** *your situation.* | | | | | | | |  |
| --- | --- | --- | --- | --- | --- | --- | --- | --- | --- |
|  | | Never | Seldom | Rather seldom | Some-  times | Often | Very often | Always | |
| 1. I feel **healthy** | | 1 | 2 | 3 | 4 | 5 | 6 | 7 | |
| 2. I am **happy** to be the person I am | | 1 | 2 | 3 | 4 | 5 | 6 | 7 | |
| 3. I am **satisfied** with my condition | | 1 | 2 | 3 | 4 | 5 | 6 | 7 | |
| 4. I can **handle the situation** I live in | | 1 | 2 | 3 | 4 | 5 | 6 | 7 | |
| 5. I **love my body** the way it is | | 1 | 2 | 3 | 4 | 5 | 6 | 7 | |
| 6. I feel **sad** | | 1 | 2 | 3 | 4 | 5 | 6 | 7 | |
| 7. I feel **physically good** | | 1 | 2 | 3 | 4 | 5 | 6 | 7 | |
| 8. I have my life **in my own hands** | | 1 | 2 | 3 | 4 | 5 | 6 | 7 | |

**PART II PHYSICAL ACTIVITY**

| ***Question 1a.*** | *During the last 7 days, on* ***how many days*** *did you perform the following types of physical activity? Think about the activities that you did for* ***at least 10 minutes at a time****. Please indicate* ***how much time*** *you do these physical activities* ***per day*** *(no week totals).* |
| --- | --- |
| 1. **Vigorous physical activities**? These are activities that take hard physical effort and make you breathe much harder than normal, like heavy lifting, digging, aerobics, or fast bicycling for ***at least 10 minutes at a time.***   **________ days per week ____ hours ___ minutes /day** | |
| 1. **Moderate physical activities**? These are activities that take moderate physical effort and make you breathe somewhat harder than normal, like carrying light loads, bicycling at a regular pace, or doubles tennis for ***at least 10 minutes at a time.*** *Please do not include walking.*   **________ days per week ____ hours ___ minutes /day** | |
| 1. **Walking?** Think about walking at work and at home, walking to travel from place to place, and any other walking that you might do solely for recreation, sport, exercise, or leisure for ***at least 10 minutes at a time.***   **________ days per week ____ hours ___ minutes /day** | |

| ***Question 1b.*** | *How much time did you spend* ***sitting*** *during* ***the last 7 days****?*  *This may include time spent sitting at a desk, visiting friends, reading, or sitting/lying down to watch television.* |
| --- | --- |
| 1. During the last 7 days, how much time did you spend sitting on a **weekday**?   **____ hours ___ minutes /day** | |
| 1. During the last 7 days, how much time did you spend sitting on a **weekend day**?   **____ hours ___ minutes /day** | |

| ***Question 2.*** | *Who supports you in your physical activity?*  *Please specify how often relatives, friends and colleagues do the following activities. Please provide an answer for each group.* | | | | | | | |
| --- | --- | --- | --- | --- | --- | --- | --- | --- |
|  | | | Never | Seldom | Some-  times | Often | Very often |  |
| 1. How often **do.... sport or exercise with you**? | | relatives | 1 | 2 | 3 | 4 | 5 |  |
|  |  | friends | 1 | 2 | 3 | 4 | 5 |  |
|  |  | colleagues | 1 | 2 | 3 | 4 | 5 |  |
| 1. How often **do.... encourage you** to be physically active? | | relatives | 1 | 2 | 3 | 4 | 5 |  |
|  |  | friends | 1 | 2 | 3 | 4 | 5 |  |
|  |  | colleagues | 1 | 2 | 3 | 4 | 5 |  |

| ***Question 3.*** | *How confident are you to be physically active?*  *Physical activity includes all activities that are at least of moderate intensity (e.g. brisk walking, jogging, cycling, swimming,…). Please circle the number that indicates how much confidence you have in the following situations that you may be physically active?* | | | | | |
| --- | --- | --- | --- | --- | --- | --- |
| How confident are you to be physically active ... | | Not confident at all | Little confident | Moderately confident | Very confident | Enormously confident |
| 1. ... if you are **tired**? | | 1 | 2 | 3 | 4 | 5 |
| 1. ... if you are in a **bad mood**? | | 1 | 2 | 3 | 4 | 5 |
| 1. ... if you are on **holidays**? | | 1 | 2 | 3 | 4 | 5 |
| 1. ... if it **rains**? | | 1 | 2 | 3 | 4 | 5 |
| 1. ... if you perceive **lack of time**? | | 1 | 2 | 3 | 4 | 5 |

| ***Question 4.*** | *Why do/don’t you engage in physically activity?*  *Using the 1-5 scale below, please indicate to what extent each of the following items is true for you.* | | | | | |
| --- | --- | --- | --- | --- | --- | --- |
|  | | Not true at all | Not true | Sometimes true | True | Very true |
| 1. I exercise because **other people say I should**. | | 1 | 2 | 3 | 4 | 5 |
| 1. **I feel guilty** when I don’t exercise. | | 1 | 2 | 3 | 4 | 5 |
| 1. **I value the benefits** of exercise. | | 1 | 2 | 3 | 4 | 5 |
| 1. I exercise because it’s **fun**. | | 1 | 2 | 3 | 4 | 5 |
| 1. **I don’t see why** I should have to exercise. | | 1 | 2 | 3 | 4 | 5 |
| 1. It’s **important to me** to exercise regularly. | | 1 | 2 | 3 | 4 | 5 |
| 1. I take part in exercise because my **friends/family/partner say I should**. | | 1 | 2 | 3 | 4 | 5 |
| 1. **I can’t see why** I should bother exercising. | | 1 | 2 | 3 | 4 | 5 |
| 1. I feel like a **failure** when I haven’t exercised in a while. | | 1 | 2 | 3 | 4 | 5 |
| 1. I find exercise a **pleasurable activity**. | | 1 | 2 | 3 | 4 | 5 |
| 1. I get **pleasure and satisfaction** from participating in exercise. | | 1 | 2 | 3 | 4 | 5 |
| 1. I think exercising is a **waste of time**. | | 1 | 2 | 3 | 4 | 5 |

| ***Question 5.*** | *What is your overall experiences with exercise?*  *The following statements refer to your overall experiences in exercise as opposed to any particular situation. Using the 1-5 scale below, please indicate the extent to which you agree with these statements by circling one number for each statement. If ‘not applicable’ for you, please circle ‘1= I don’t agree at all’.* | | | | | | |
| --- | --- | --- | --- | --- | --- | --- | --- |
|  | | I don’t agree at all | I agree a little | I somewhat agree | I agree a lot | I completely agree |  |
| 1. I feel I have made a lot of **progress** in relation to the goal I want to achieve. | | 1 | 2 | 3 | 4 | 5 |  |
| 1. The way I exercise is in agreement with **my choices and interests**. | | 1 | 2 | 3 | 4 | 5 |  |
| 1. I feel I perform **successfully** the activities of my exercise program. | | 1 | 2 | 3 | 4 | 5 |  |
| 1. My relationships with the people I exercise with are very **friendly**. | | 1 | 2 | 3 | 4 | 5 |  |
| 1. I feel that the way I exercise is **the way I want to**. | | 1 | 2 | 3 | 4 | 5 |  |
| 1. I feel exercise is an activity which **I do very well**. | | 1 | 2 | 3 | 4 | 5 |  |
| 1. I feel I have **excellent communication** with the people I exercise with. | | 1 | 2 | 3 | 4 | 5 |  |
| 1. I feel that the way I exercise is a true **expression** of who I am. | | 1 | 2 | 3 | 4 | 5 |  |
| 1. I am able to **meet the requirements** of my exercise program. | | 1 | 2 | 3 | 4 | 5 |  |
| 1. My relationships with the people I exercise with are **close**. | | 1 | 2 | 3 | 4 | 5 |  |
| 1. I feel that I have the **opportunity** to make choices with regard to the way I exercise | | 1 | 2 | 3 | 4 | 5 |  |

| ***Question 1.*** | *How do you feel at work?*  *Please indicate how often each statement applies to you by circling the number (from 0 to 6) that best fits your situation.* | | | | | | | |
| --- | --- | --- | --- | --- | --- | --- | --- | --- |
|  | | Never | Almost never (≤1x/year) | Rarely  (≤1x/month) | Sometimes  (≥2x/month) | Often  (≤1x/week) | Very often  (≥2x/week) | Always (daily) |
| 1. At my work, I feel bursting with **energy** | | 0 | 1 | 2 | 3 | 4 | 5 | 6 |
| 2. At my job, I feel **strong and vigorous** | | 0 | 1 | 2 | 3 | 4 | 5 | 6 |
| 3. I am **enthusiastic** about my job | | 0 | 1 | 2 | 3 | 4 | 5 | 6 |
| 4. My job **inspires** me | | 0 | 1 | 2 | 3 | 4 | 5 | 6 |
| 5. When I get up in the morning, I **feel like** going to work | | 0 | 1 | 2 | 3 | 4 | 5 | 6 |
| 6. I feel **happy** when I am working intensely | | 0 | 1 | 2 | 3 | 4 | 5 | 6 |
| 7. I am **proud** on the work that I do | | 0 | 1 | 2 | 3 | 4 | 5 | 6 |
| 8. I am **immersed** in my work | | 0 | 1 | 2 | 3 | 4 | 5 | 6 |
| 9. I get **carried away** when I’m working | | 0 | 1 | 2 | 3 | 4 | 5 | 6 |

**PART III WORK**

| ***Question 2.*** | *What does mean to you?*  *  *Please indicate to what extent you agree with the following statements by circling the number (from 1 to 5) that best fits your situation.* | | | | | |  |
| --- | --- | --- | --- | --- | --- | --- | --- |
|  | | Totally disagree | Disagree | (Dis)agree  a little | Agree | Totally agree | |
| 1. takes a **very important place in my life**  * | | 1 | 2 | 3 | 4 | 5 | |
| 2. **means a lot** to me  * | | 1 | 2 | 3 | 4 | 5 | |
| 3. I feel **strongly connected** to  * | | 1 | 2 | 3 | 4 | 5 | |
| 4. I feel a **close bond** with  * | | 1 | 2 | 3 | 4 | 5 | |
| 5. I am **proud** that I am an employee of  * | | 1 | 2 | 3 | 4 | 5 | |

* Name of the company
